# Indoleamine 2,3-Dioxygenase and Tolerance: Where Are We Now?

**DOI:** 10.3389/fimmu.2017.01360

**Published:** 2017-10-27

**Authors:** Andrew L. Mellor, Henrique Lemos, Lei Huang

**Affiliations:** ^1^Faculty of Medical Sciences, Institute of Cellular Medicine, Newcastle University, Newcastle upon Tyne, United Kingdom

**Keywords:** indoleamine 2,3-dioxygenase, tolerance, autoimmunity, nociceptive pain, transplant, transplantation immunology

## Abstract

Cells expressing IDO suppress innate and adaptive immunity to promote tolerance by catabolizing the amino acid tryptophan (Trp) and other indole compounds. Interferon type I (IFN-I) and type II (IFN-II) produced at sites of inflammation or by activated immune cells are potent IDO inducers because mammalian IDO genes contain IFN response elements. Elevated IDO expression by dendritic cells (DCs) is of particular significance because IDO activity converts mature DCs into tolerogenic APCs that suppress effector T cells (Teff) and promote regulatory T cells (Tregs), thereby promoting tolerance. Local Trp depletion and production of immune suppressive Trp catabolites contribute to tolerogenic processes by activating metabolic pathways responsive to amino acid withdrawal and aryl hydrocarbon signaling, respectively. Sustained IDO elevation creates local immune privilege that protects tissues from immune-mediated damage and allows tissues to heal. This response occurs in lymphoid tissues when DNA released by dying tissue cells is sensed to induce specialized DC subsets to acquire tolerogenic phenotypes. The tolerogenic effects of IDO also promote tumorigenesis and help establish immune checkpoints in cancer, as malignant cells are protected from immune surveillance. Similar processes may attenuate host immunity to some pathogens that persist in immunocompetent individuals. However, if inflammation with IDO involvement is not resolved, chronic immune activation at such sites causes progressive tissue damage over time. Another effect of sustained IDO activity is enhanced pain sensitivity, as some Trp catabolites produced by cells expressing IDO are neuroactive. In this review, we summarize links between IDO and chronic inflammatory diseases and discuss prospects for exploiting IDO and Trp catabolism to suppress immunity and promote tolerance for clinical benefit, with particular emphasis on protecting tissues from destructive autoimmunity.

## Introduction

Higher mammals possess two closely linked and homologous genes encoding indoleamine 2,3-dioxygenase (IDO1, IDO2), which catabolizes compounds containing indole rings, including tryptophan (Trp) and the neurotransmitter serotonin, aka 5-hydroxytryptamine (5HT). IDO1 gene expression is responsive to interferons (IFNs), explaining why IDO activity is elevated in many inflammatory settings, including infectious, allergic and autoimmune (AI) diseases, tumorigenesis, and pregnancy. In this brief review, we highlight key aspects of IDO1 immunobiology and summarize prospects for exploiting this pathway to protect tissues from immune-mediated damage in patients with AI syndromes and transplanted tissues. The concise format of this review precludes a comprehensive overview and readers are directed elsewhere for detailed discussions of the current state of this highly active field. The ability of cells expressing IDO1 genes to suppress immune responses was first described almost 20 years ago (1). Since then, research on IDO has blossomed and IDO inhibitors are promising immune checkpoint inhibitor drugs to enhance cancer immunotherapy (2, 3).

## IFNs, IDO, and Immune Balance

Interferons released at sites of inflammation stimulate hundreds of downstream genes, known collectively as IFN-stimulated genes (ISGs). Historically, ISGs that activate immune cells to incite immunity to infections have been the major focus of research. However, some ISGs activate immune regulatory cells to promote tolerogenic, rather than immunogenic processes. The local balance of immunogenic and tolerogenic responses to IFNs is a key factor driving the effects of local inflammation on immune responses and tissue functions.

IDO1 is an example of a tolerogenic ISG because IDO suppresses immune responses and the IDO1 gene is responsive to both IFN type I (IFN-I) and type II (IFN-II). IFN response elements called ISRE and GAS sequences, which confer responsiveness to IFN-I and IFN-II signaling, respectively, are located in mammalian IDO1 gene promoters. Though most cell types express IFN receptors, IFNs induce IDO only in select cell types because IFN signaling is regulated in some cells. For example, discrete subsets of dendritic cells (DCs) from humans and mice are competent to express IDO when exposed to IFNs (4). Importantly, IDO1 gene transcription may not enhance IDO enzyme activity due to posttranslational controls, such as limited access to hemin, an IDO enzyme co-factor, local redox status, and nitric oxide, which inhibits heme/O_2_ conjugation needed to break indole structures (5, 6). IDO can also promote tolerance *via* non-catalytic signaling to induce TGFβ release by some DCs (7). Thus, whether IDO activity manifests in inflamed tissues depends on many factors, linked to the complexity of biochemical pathways and cell interactions driving responses to IFNs in tissues. Thus, it is important to study responses to IFNs in physiologic settings, where multiple effects are integrated to generate particular responses to inflammatory insults. Though much can be learned by studying how cultured or physiologic cells and isolated tissues respond to IFNs, these approaches cannot predict physiologic outcomes accurately in the absence of the full panoply of biochemical processes and cellular interactions that exist in inflamed tissues.

## IDO and Trp Catabolism

**Figure 1:**
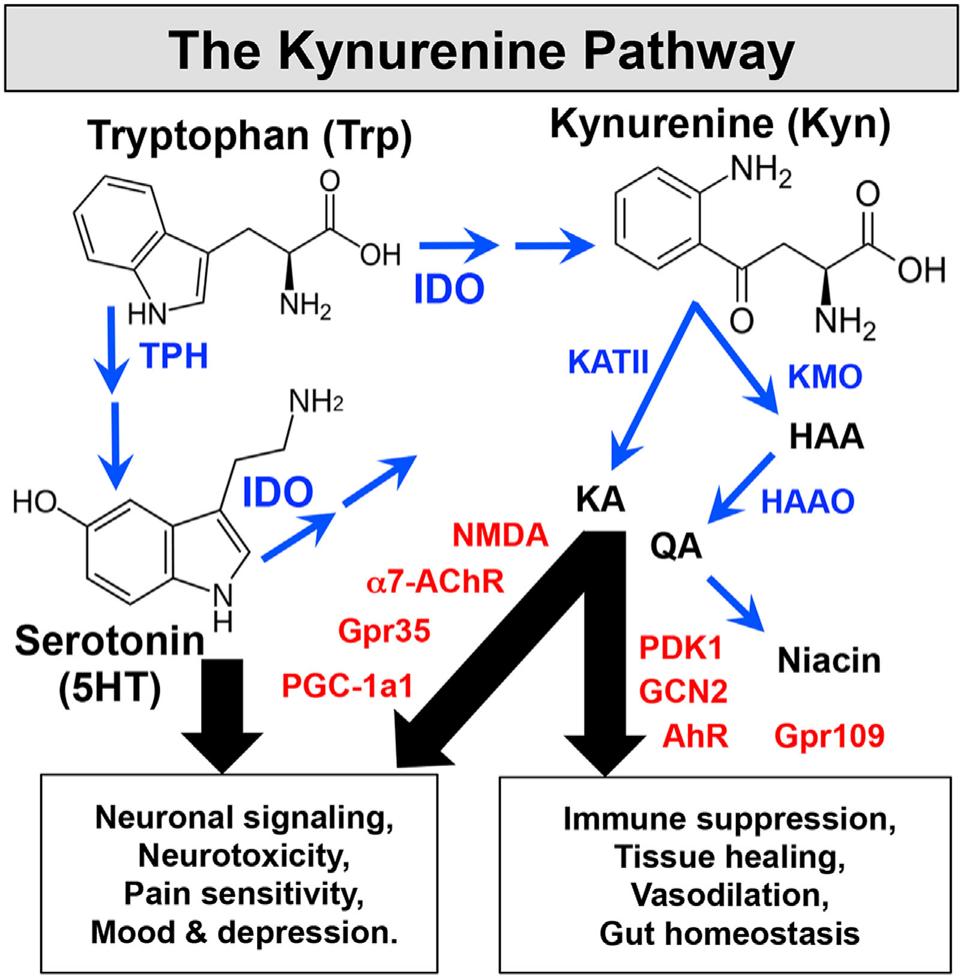
The Kynurenine pathway. IDO catabolizes indole compounds, including Trp and 5-hydroxytryptamine. Enzymes (blue) downstream of IDO generate bioactive catabolites called kynurenines. Immune and CNS cells express sensors (red) that detect these metabolic changes to impact immunologic and neurologic processes.

IDO catalyzes the initial, rate-limiting step in oxidative catabolism of compounds containing indole rings (Figure 1), including Trp and 5HT. Cells expressing IDO1 genes deplete Trp and generate bioactive catabolites, known as kynurenines, after the catabolite kynurenine (Kyn). Enzymes downstream of IDO further degrade Kyn to generate kynurenic acid (KA), 3-hydroxy-anthranilic acid (HAA), quinolinic acid (QA), niacin, and other catabolites. Some immune cells can sense Trp depletion or catabolites to suppress innate and adaptive immunity and promote tolerogenic responses. Limiting access to Trp activates the ribosomal kinase GCN2, which senses binding of uncharged tRNA to ribosomes. Activated GCN2 triggers the integrated stress response (ISR) to amino acid withdrawal, which induces CHOP gene expression but shuts down most gene transcription to promote cell autophagy. The ISR blocks cell cycle entry by TCR-activated T cells and activates resting Foxp3-lineage regulatory CD4 T cells (Tregs) to promote tolerogenic responses to inflammatory signals from immune adjuvants and tumor vaccines (8). Some catabolites in the Kyn pathway bind to receptors expressed by immune cells to promote tolerogenic responses. For example, HAA, Kyn, and KA suppress T cell responses by binding to PDK1 or aryl hydrocarbon receptors (AhR) in T cells, APCs, or other immune cells. As well as influencing inflammatory and immunologic processes, the Kyn pathway also drives neurologic comorbidities such as pain, depression, and fatigue, commonly associated with chronic inflammatory disease. By consuming Trp, the substrate for 5HT and melatonin synthesis, IDO impacts mood and promotes depression. Moreover, receptors such as NMDA, α7AChR, and Gpr35 expressed by neuronal cells can sense neuroactive catabolites such as KA and QA. An intriguing link between physical exercise and reduced depression was reported due to increased uptake of circulating Kyn by PGC1a1 receptors expressed by active skeletal muscles (9). Niacin, another product of the kynurenine pathway that binds to the Gpr109a receptor, also suppresses colonic inflammation and carcinogenesis to promote gut homeostasis and health (10). Collectively, these points reveal pivotal roles for the Kyn pathway as a critical modifier of immunologic and neurologic responses to inflammation *via* the biochemical effects of catabolizing indole compounds such as Trp and 5HT.

## Chronic Inflammatory Disease and IDO

Elevated IDO activity manifests in many chronic inflammatory syndromes, including cancer, infections, AI and allergic diseases, transplant rejection, and pregnancy (11). A link between IDO and immune regulation was first described in pregnancy. IDO inhibitors applied to pregnant mice induced allogeneic fetal rejection by maternal T cells, while sparing syngeneic fetus’ from the same fate (1), indicating that IDO stops maternal T cells from attacking fetal allografts during pregnancy. Further studies revealed that complement activation drove fetal allograft rejection when IDO was inhibited, indicating that IDO blocks complement activation by maternal T cells responsive to fetal alloantigens (12).

### IDO and Hypo-Immune Syndromes

IDO expression is often elevated in inflamed tumor microenvironments in mice and humans. Tumor cells or tumor-associated cells in malignant lesions may express IDO. Elevated IDO may also manifest in local lymph nodes draining sites of tumor growth. The paradigm that IDO promotes tolerogenic responses suggests that elevated IDO activity during tumorigenesis contributes to robust tumor resistance to natural and vaccine-induced anti-tumor immunity. Consistent with this notion, IDO inhibitors enhance tumor immunity to retard tumor growth in several mouse models, identifying IDO as a cancer immune checkpoint. Consequently, IDO inhibitors are under scrutiny as potential immune checkpoint inhibitor drugs in ongoing clinical trials in cancer patients. Early indications are that IDO inhibitors are well tolerated and may enhance clinical response rates to some cancers. However, heightened risk of inciting autoimmunity may be an undesirable side effect, as for other immune checkpoint inhibitors. Similarly, IDO induced by infectious pathogens may attenuate host immune responses. Most infections induce rapid IDO upregulation since IFN-I is a common innate response to many bacterial, viral, fungal, and parasitic infections. Largely, this is because Toll-like receptors (TLRs) and nucleic acid sensors that recognize pathogen-associated molecular patterns stimulate IFN-I production at sites of infection. Some pathogens that cause chronic infections may exploit the Kyn pathway to promote persistence in immunocompetent individuals. Consistent with this paradigm, IDO inhibitors reduced *Leishmania major* burdens in mice, even when applied at the peak of infection (13, 14). IDO inhibitors also reduced lentiviral HIV-1 burdens in a mouse model of HIV-1 encephalitis, suggesting that robust IDO induction on HIV-1 infection attenuates host immunity (15). However, opposing protective effects of IDO for hosts and pathogens were observed in distinct models of infection in mice. For example, IDO inhibition induced uniform mortality of mice infected with the parasite *Toxoplasma gondii* (14), consistent with earlier work showing that IDO contributes to innate host resistance to some pathogens such as parasitic Toxoplasma and fungal Candida infections. Moreover, despite >100-fold increase in lung IDO activity during influenza infections in mice, IDO ablation had no impact on virus burdens and only nuanced effects on host T cell responses to influenza infection (16). Thus, interventions to boost host immunity to pathogens by manipulating the Kyn pathway must be investigated thoroughly to avoid potential undesirable consequences.

### IDO and Hyper-Immune Syndromes

Elevated IDO activity also manifests in many AI and allergic syndromes in mice and humans. IDO attenuates AI progression in several mouse models of type I diabetes, multiple sclerosis (MS), rheumatoid arthritis (RA), systemic lupus erythematosus, and graft-versus-host disease since IDO1 gene ablation potentiated disease onset and severity mediated by T cells, while enhancing IDO1 gene expression attenuated disease progression and severity (2). One exception is the KxBN arthritis model driven by AI B cells, potentially because IDO and IL6 (aka B cell growth factor) co-promote B cell autoimmunity in this model (17). IDO may also have equivocal roles in allergic diseases, as T_h_2-mediated allergic airway inflammation in mice was attenuated by treating mice with TLR9 ligands (CpGs) to induce IDO and by ablating IDO1 genes (18, 19). The reason for this discrepancy is unknown, though distinct cell types expressing IDO may promote or attenuate allergic disease. This point is important when considering potential roles for IDO in immune responses, as several cell types may express IDO and mediate diametric effects on immune responses in particular inflammatory settings.

## Using IDO Inducers to Promote Tolerance

The tolerogenic effects of cells expressing IDO have prompted efforts to boost IDO activity, particularly in DCs, as novel strategies to alleviate AI disease and prolong transplant survival. A number of strategies can be envisaged to exploit these properties for clinical benefit, as described below.

### IDO1 Gene Transduction

In transplantation, proof-of-principle emerged from several studies on rodent models of lung engraftment showing that pre-transplant IDO1 gene transduction promoted robust lung allograft survival in the absence of global immune suppressants (20–22). Moreover, CD8^+^ T cells infiltrating IDO1-transduced lungs exhibited impaired effector functions due to selective IDO-mediated inhibition of mitochondrial electron transfer (20), a finding reminiscent of functional impairment of tumor-infiltrating T cells in cancer. It is unclear from these reports if lung allograft survival was dependent on sustained IDO activity to suppress alloimmunity or if stable tolerance to donor alloantigens not requiring sustained IDO1 gene expression was induced post-transplantation. Nevertheless, as in pregnancy, these studies reveal that elevated IDO activity protects healthy tissues from immune-mediated destruction. However, as gene transduction in clinical settings is complicated technically and raises serious risks, a key question is how to elevate and, as may be necessary, to sustain IDO in order to protect healthy tissues in patients.

### Soluble CTLA4

A potential alternative strategy is to use drugs to induce IDO. Several IDO inducing drugs are known, including soluble CTLA4, TLR ligands, and DNA. The immunosuppressant drug Abatacept (Orencia™) is a soluble CTLA4 molecule that blocks CD28-mediated T cell co-stimulation. Abatacept is used to alleviate RA in some patients and may help alleviate other AI syndromes, though treatments are expensive. In mice, soluble CTLA4 reagents induced specialized DC subsets to express IDO1 genes and acquire potent tolerogenic functions *via* “reverse signaling” mediated by CD80/86 (B7) surface ligands on DCs that bind CD28 and CTLA4 on T cells (23, 24). It is unclear how B7 molecules stimulate DCs to express IDO1 genes but IFN-I signaling and critical functions in the Fc (Ig) domain of soluble CTLA4 were required for this response. Commercial products like Orencia were optimized for CD28 binding and bio-availability. Consequently, these reagents may have lost the ability to induce IDO if critical Fc (Ig) domain functions were engineered out. Reverse signaling *via* other ligand-receptor pathways (e.g., GITR, ICOS, CD200) to induce DCs to express IDO has also been shown to cause DCs to adopt tolerogenic phenotypes (25–27).

### TLR Ligands

TLR4 (LPS) and TLR9 (CpGs) ligands also induce DCs to express IDO. Thus, CpGs given systemically to mice activated resting Tregs to suppress effector T cell responses *via* a mechanism dependent on IDO1 gene expression by discrete splenic DC subsets. In contrast, CpG treatments induced resting Tregs to convert into T_h_17 T cells when IDO1 genes were ablated, identifying DCs as pivotal regulators of tolerogenic and immunogenic responses to TLR9 ligands contingent on if DCs were induced to express functional IDO or not, respectively. However, co-induced pro-inflammatory and consequent immunogenic responses to TLR ligands may overcome tolerogenic responses, especially in inflamed tissues.

### Stimulator of IFN Genes (STING) Agonists

Systemic treatments with DNA nanoparticles (DNPs) induced IDO in many mouse tissues. In spleen, myeloid DCs ingested DNPs and sensed cargo DNA to activate the STING adaptor, a potent IFN-I inducer (28, 29). Consistent with these findings DNPs alleviated AI disease progression in mouse models of RA and MS (EAE) and therapeutic responses to DNPs depended on STING-IFN-I signaling to induce IDO in DCs (28, 30). Moreover, synthetic cyclic dinucleotides (CDNs) that mimic natural STING agonists generated by the cytosolic DNA sensor cyclic guanyl-adenyl diphosphate (cGAMP) synthase (cGAS) also alleviated AI syndromes in mice (30). Paradoxically, CDNs administered directly into developing tumors stimulated potent anti-tumor immunity that attenuated tumor growth in mice (31). Diametric responses to CDNs in AI and tumor models emphasize the critical importance of dosing since systemic and intra-tumoural delivery of CDNs was required to induce tolerogenic and immunogenic responses, respectively, in these distinct inflammatory settings.

### Interferons

In principle, IFN-I could also be used to induce IDO1-dependent tolerogenic responses since IFN-I mediated such responses to B7, TLR, and STING ligands. Indeed, some therapeutic effects of IFN-I (IFNβ) in MS patients may accrue from tolerogenic responses mediated *via* IDO, though it is unclear if this does occur. As with TLR ligands, undesirable pro-inflammatory (immunogenic) responses to IFN-I (or IFN-II) may predominate and overcome tolerogenic responses mediated by IDO, making the clinical use of IFNs to induce tolerance high risk.

## IDO Pain and Depression

As stated above, the Kyn pathway has profound effects on neurologic, as well as inflammatory and immunologic, processes. The KA:QA balance is a major factor in neuro-inflammatory syndromes, as high QA levels correlate with neuro-toxicities and dementia, while KA ameliorates these damaging effects. Heightened and sustained IDO activity during chronic inflammatory diseases may contribute to enhanced pain sensitivity, depression, and fatigue, which are common comorbidities associated with many of these syndromes. These effects complicate efforts to exploit the Kyn pathway because beneficial therapeutic effects that suppress hyper-immunity and promote tolerance may come at the cost of increased pain, depression and fatigue, as consequences of therapy. Nevertheless, using IDO inducers to reduce disease-associated inflammation and hyper-immunity in the short term may attenuate innate IDO activity that promotes debilitating neurologic comorbidities. For example, in the EAE model of MS in mice, IDO activity was elevated in CNS tissues during EAE induction but STING agonists treatments to induce IDO in peripheral lymphoid tissues and suppress autoimmunity abolished IDO1 expression in the CNS (30). Thus, overall beneficial effects in attenuating disease progression and reducing comorbidities may accrue from the diametric effects of IDO activity in distinct neuronal and lymphoid tissues. Furthermore, it may be possible to use drugs that modify the Kyn pathway to enhance production of immune suppressive Trp catabolites and reduce production of neurotoxic Trp catabolites. Thus, using HAAO inhibitors to block neurotoxic QA production and KATII inhibitors to promote immune suppressive HAA production may enhance therapeutic responses and reduce neurologic comorbidities, especially if combined with reagents that stimulate the Kyn pathway.

## Summary and Future Prospects

Elevated IDO activity manifests in many settings of high clinical significance, driving substantial interest in manipulating IDO and the Kyn pathway for clinical benefit. Extensive research on mouse models of chronic inflammatory syndromes supports the hypothesis that IDO activity suppresses innate and adaptive immunity by depleting Trp and generating bioactive Trp catabolites. Though IDO-mediated tolerogenic responses to sustained inflammation are particularly prominent, elevated IDO activity may also manifest rapidly to overcome co-induced immunogenic responses. It remains to be seen how many observations from mouse models of clinical syndromes can be translated into clinical practice. Promising initial results from recently completed or ongoing experimental clinical trials on cancer patients using several different IDO inhibitors will be the first potential clinical application to be tested fully. If outcomes support the use of IDO inhibitors in cancer, future research will focus on identifying optimal drug combinations to halt tumor growth and disrupt immune checkpoints in the widest range of patients, without stimulating autoimmunity or other toxic responses. Since immune checkpoints may also promote pathogen persistence and many infections induce IDO activity, there may also be opportunities to use IDO inhibitors to reduce pathogen burdens and promote pathogen clearance in patients with chronic infections. However, more research is needed to understand the balance between anti-microbial and (host) tolerogenic effects of IDO, as the risk of inducing undesirable toxic effects is high until the role of IDO in specific chronic infections is elucidated fully. On the flip side, enhancing IDO activity as novel strategies to prevent or alleviate hyper-immune syndromes are supported by many proof-of-concept studies in mouse models of transplantation and AI syndromes. Translating these outcomes into clinical application will take some time, in part due to potential for many IDO inducing drugs to elicit tissue-damaging immunogenic responses as well as co-inducing tissue-protective tolerogenic responses. Thus, it may not be straightforward to satisfy ethical and corporate requirements for specific molecular targets driving unequivocal outcomes, unless more precise ways are found to manipulate the immune balance for clinical benefit. Finally, the combined immunologic and neurologic effects of bioactive Trp catabolites emphasizes the key roles of IDO and the Kyn pathway as drivers or regulators of many chronic conditions and common comorbidities, including pain depression and fatigue. Clearly, while more research is needed to understand the complex roles of IDO and the Kyn pathway in chronic diseases, the prospects are good for major novel insights and advances leading to clinical practice.

## Author Contributions

AM wrote the review. HL and LH read and critiqued the review.

## Conflict of Interest Statement

The author (AM) is a consultant for NewLink Genetics Inc. and receives income from this source. All other authors have no competing interests to disclose.
